# Attentional influences on functional mapping of speech sounds in human auditory cortex

**DOI:** 10.1186/1471-2202-5-24

**Published:** 2004-07-21

**Authors:** Jonas Obleser, Thomas Elbert, Carsten Eulitz

**Affiliations:** 1 Department of Psychology, University of Konstanz, Germany; 2 Department of Linguistics, University of Konstanz, Germany; 3 Department of Psychiatry and Psychotherapy, School of Medicine, University of Aachen, German

## Abstract

**Background:**

The speech signal contains both information about phonological features such as place of articulation and non-phonological features such as speaker identity. These are different aspects of the 'what'-processing stream (speaker vs. speech content), and here we show that they can be further segregated as they may occur in parallel but within different neural substrates. Subjects listened to two different vowels, each spoken by two different speakers. During one block, they were asked to identify a given vowel irrespectively of the speaker (phonological categorization), while during the other block the speaker had to be identified irrespectively of the vowel (speaker categorization). Auditory evoked fields were recorded using 148-channel magnetoencephalography (MEG), and magnetic source imaging was obtained for 17 subjects.

**Results:**

During phonological categorization, a vowel-dependent difference of N100m source location perpendicular to the main tonotopic gradient replicated previous findings. In speaker categorization, the relative mapping of vowels remained unchanged but sources were shifted towards more posterior and more superior locations.

**Conclusions:**

These results imply that the N100m reflects the extraction of abstract invariants from the speech signal. This part of the processing is accomplished in auditory areas anterior to AI, which are part of the auditory 'what' system. This network seems to include spatially separable modules for identifying the phonological information and for associating it with a particular speaker that are activated in synchrony but within different regions, suggesting that the 'what' processing can be more adequately modeled by a stream of parallel stages. The relative activation of the parallel processing stages can be modulated by attentional or task demands.

## Background

This study explores attentional modulation within the 'what'-stream of the auditory modality during phoneme processing. Knowledge of speech sound representation in the auditory domain is still sparse. However, parallels to the extensively studied visual modality and also to the somatosensory domain are becoming evident. For example, columnar mapping of several stimulus properties (as known from the visual cortex) has been revealed in human and animal research: acoustic parameters like spectral bandwidth, periodicity, stimulus intensity [1,2] or – for human speech sounds – distance between spectral peaks [3,4] appear to be mapped perpendicularly to the main cochleotopic gradient. Recently, a segregation of a ventral 'what' and a dorsal 'where' stream – as long established in the visual system [5] – has also been proposed for the auditory system. This conclusion was based on neuroanatomical and functional studies in macaques [6-8] and has been substantiated in humans [9,10].

Given these parallels between sensory domains and the increasing preference for complex stimuli along the auditory central pathway, more complex topologies such as language-specific maps in auditory cortex are also plausible, and evidence for individually ordered mapping of speech sounds is growing [11-15] (for speech-specific vocalizations in animals see [8,16]). More specifically, data from our lab imply map dimensions along phonological features which build the basic components of speech sounds: In Obleser et al. [15], responses to DORSAL vowels (which are articulated with the back of the tongue and which exhibit a small distance between spectral peaks, i.e., small F_1_-F_2 _distance) were located more posterior in auditory association cortex than responses to CORONAL vowels (which are articulated with the tip of the tongue and which exhibit a large distance between spectral peaks, i.e., larger F_1_-F_2 _distance), and a topographical shift between these classes of vowels even when embedded in non-words has been reported [15,17].

Research has long been tackling the question of attention and attentional top-down modulation that may tune cortical neurons and with it functional maps in a context-specific manner: In the visual domain, a top-down influence on receptive fields of areas as basic as VI has been shown [18,19], and in the somatosensory domain Ergenzinger and colleagues reported that drastic changes in functional maps can be experimentally induced even on a thalamic level [20]. The thalamic homuncular representation of a monkey's hand becomes blurred and distorted when top-down modulation from somatosensory cortex is blocked neurochemically within the cortex. These results emphasize the possibility of attention-dependent modulation of maps, a topic exemplified in a somatosensory MEG mapping study by Braun and colleagues [21]: In a somatosensory stimulation with small brushes moving back and forth across the digit tips, subjects either attended the movement of single brushes on single digits and reported the movement direction or they attended and reported the global direction of all brushes on all five digits. Magnetic source imaging of the somatosensory evoked field revealed a typical homuncular representation of the single digits spread along the post central gyrus only in the condition where the focus of attention was on single digits rather than on the hand as a whole. In the latter condition, top-down attentional demands temporarily seemed to blur the single digit mapping.

For the developing field of speech sound mapping, top-down influences of attentional demands on functional organization at the different stages in the processing streams have not been sufficiently studied. Nevertheless, it becomes a central issue when the functional architecture of the effortless and robust perception of speech shall be understood. It is common to study speech perception either in passive oddball paradigms [22,23] where the subject's attention is deliberately forced to a movie or to reading a book, or in passive listening conditions where no attentional control is experimentally induced (e.g. [24,25]), or in active target detection tasks where the attention is commonly focused on the phonological content of the speech material [14,15,26].

We analyzed the magnetic N100 (N100m) response to two vowels [o] and [ø], both produced by a male and a female speaker. Subject's attention was either on the vowel or on the speaker difference, in a counterbalanced order. How would a controlled shift of attention from specific phonological features of speech to features of speaker identity affect the speech sound mapping in timing and topography of the brain response? Two concurrent outcomes are conceivable here: First, from the numerous parallels between the auditory and other sensory domains, one might expect a blurring of differences of the phonological map in auditory cortex when features such as the speaker identity rather than phonological differences are attended over minutes. Second, phonological processing could be the default process needed in all speech-listening situations and should therefore activate phonological feature maps irrespectively of attentional demands. We would then expect that the separate mapping of DORSAL and CORONAL vowels described previously [15] is unaffected by an attentional focus on speaker identity. However, a shift of activational patterns as an entity would reveal more about the staging of parallel processing in the flow of the 'what' stream.

## Results

In 21 of 22 subjects, a clear waveform deflection around 100 ms post vowel onset was observed (Fig. 2) in all conditions over both hemispheres and sensor space parameters peak latency and amplitude were obtained. Satisfying and physiologically plausible dipole fits (see methods) in both hemispheres could be obtained in 17 subjects and were subjected to statistical analysis.

### N100m latency, amplitude and source strength

Analysis of the N100m root mean square (RMS) peak latency revealed foremost a main effect of vowel (F_1,20 _= 44.8, p < .0001, Fig. 2), whereby the DORSAL vowel [o] consistently elicited N100m peaks 5 ms later than the CORONAL vowel [ø]. In sensor space, an enhancement of RMS peak amplitude for the [ø] vowel by 10 fT (Fig. 2) almost attained significance (F_1,20 _= 4.12, p < .06). However, the effect was significant in source space that is not influenced by varying head-to-sensor positions: The [ø] dipole source strength, an estimate for the amount of massed neuronal activity, was larger for the [ø] vowel than for the [o] by 25 % or 6 nAm (F_1,16 _= 9.36, p < .01). No hemispheric differences in signal power between vowel categories or tasks were apparent.

### N100m source location and orientation

In agreement with previous findings with a more comprehensive set of vowels [15], the vowel categories [o] and [ø] elicited statistically different centers of activity along the anterior-posterior axis (F_1,16 _= 7.73, p < .01), that is, the auditory processing in the DORSAL vowel [o] was reflected by a more posterior ECD location (Fig. 3). A difference in source configuration was also evident from a more superior position of the [o] source (F_1,16 _= 12.28, p < .01), a more vertical orientation (F_1,16 _= 5.81, p < .05) than the [ø] source, and from an angular difference between the two vowel categories in the sagittal plane (i.e. the [o] source was located more posterior and inferior, F_1,16 _= 10.91, p < .01) and in the axial plane (i.e. the [o] source was also located more posterior and lateral, F_1,16 _= 6.82, p < .05, relative to the [ø] source). None of these effects showed an interaction with hemisphere, but data gained further validity as the right-hemispheric sources were all located more posterior (F_1,16 _= 8.88, p < .01), more inferior (F_1,16 _= 4.27, p < .06) and were tilted more vertically (F_1,16 _= 14.29, p < .01) than their left-hemispheric counterpart. Such a difference is to be expected from previously reported N100 asymmetries between cerebral hemispheres [27-30].

The relative mapping of phonological features of the speech signal [14,15] was not affected by the task-induced shifts of attention. However, shifts of subjects' attentional focus from phonological categorization to identification of the speaker's voice shifted vowel sources as a whole to more posterior and superior locations within the supratemporal plane. Statistically, the speaker categorization task produced more superior (F_1,16 _= 4.72, p < .05) and marginally more posterior (F_1,16 _= 3.36, p < .10) ECD locations, which was also evident by an angular displacement in the sagittal plane (F_1,16 _= 4.6, p < .05). The effect seemed to be driven by changes in the left hemisphere but the task × hemisphere interaction never attained significance (all F < 1).

When brain responses were analyzed separately for stimuli spoken by male and female speaker, which yielded satisfying dipole solutions only in 12 subjects, the most striking finding was a consistent speaker × task interaction of the dipole location in both the sagittal plane (F_1,11 _= 10.83, p < .01) and the axial plane (F_1,11 _= 7.16, p < .03). That is, subjects' attentional focus slightly affected the relative displacement of male and female voice-evoked brain responses: In both the sagittal plane and the axial plane, a significant 4° difference emerged in the phonological categorization task (both p < .05), which vanished in the speaker categorization task. In contrast, as reported above, no such task influence was evident in the relative position of vowel-evoked brain responses.

### Performance

Overall target detection rate was 94.1 %, false alarms occurred in 5.5% of all trials. Responses of the 17 subjects whose brain responses were subjected to magnetic source imaging were analyzed in detail: The phonological categorization task (93.2 ± 3.0 % correct, 4.9 ± 2.2 % false alarms, M ± SEM) and the speaker categorization task (95.0 ± 2.9 % correct, 6.2 ± 3.2 % false alarms) did not differ significantly (one-way repeated measures ANOVAs, all F < 1).

## Discussion

This study was set up to explore potential influences of the attentional focus on the mapping of speech sounds within the auditory cortex. With subject's attention either on the phonological differences or on the speaker difference between vowel stimuli, we mapped the auditory evoked N100m and localized its sources that fitted well with a single dipole per hemisphere. All responses were located in the perisylvian region. Furthermore, the relative distribution of sources indicated an interesting pattern. As hypothesized and expected from previous studies, the fundamental location difference between the sources of the DORSAL vowel [o] source and the CORONAL vowel [ø] [15,17] could be replicated under both attentional conditions. In contrast, the corresponding difference between speaker-dependent sources was subject to task influences.

That is, a shift of subjects' attention to a non-phonological acoustic feature, the speaker identity, did not blur the spatial segregation within the speech sound map. In contrast, the [ø] and [o] generators were slightly displaced towards more posterior and more superior locations when subjects focused on speaker identity.

In most situations, a listener may automatically extract the phonological invariants from the speech signal in order to access lexical information, for example the meaning of the information inherent in speech. Speaker-dependent features such as pitch and periodicity should not play a crucial role in this phonological decoding process. This is what we mimicked by asking our subjects to detect a certain vowel in a stream of varying speech sounds. However, in cocktail-party-like situations there is the additional demand to attend acoustic properties of certain speech streams or speakers, and we implemented it by asking our subjects to detect a certain voice in a stream of varying speakers. Speaker identification comprises an important but not necessarily orthogonal process to phonological decoding in speech perception: areas in the upper bank of the superior temporal sulcus (STS) have been identified previously [31] to be voice-selective (as opposed to other environmental sounds), and in many situations the selective tracking of one voice amongst others is a prerequisite for decoding the phonological content of this speaker's utterances. The displacement of dipolar sources seen here may mirror the involvement of additional cortical areas, such as the voice-specialized part in the STS [31] or pitch-specialized areas in the primary auditory cortex. An additional STS activation would most likely elicit an inferior shift of the dipole sources during speaker categorization. However, a shift into the opposite direction was obtained. This might indicate that the contribution of the voice-specialized part of the STS around 100 ms post-stimulus onset is small compared to other additional cortical areas, such as pitch-specialized areas in the primary auditory cortex. It is now well-established that a finegrained analysis of the speech signal takes place mainly in anterior parts of the supratemporal gyrus [17,32-34], thereby anterior of primary auditory areas. Consequently, the activity shift towards more posterior sites we observed in the speaker categorization task strongly argues for an additional involvement of these primary auditory areas. Unfortunately, we cannot dissociate speaker identification processes from pitch processing in the current study. However, pitch differences are among the primary cues dissociating male and female voices, and a clear involvement of auditory core areas in pitch processing has been shown in a recent MEG study focusing on pitch detection mechanisms [35].

## Conclusions

Data presented here suggest that the systematic mapping of speech sounds within the auditory cortex is robust under changing attentional demands and not tied to phonological awareness. However, the general shift of activity when a non-phonological speaker categorization must be accomplished shows that speech sound representations are modulated in their locations in a context-dependent manner. Situational demands obviously influence the differential but time-synchronous involvement of specialized neuronal assemblies that contribute to speech sound decoding in a top-down fashion. Hence, the spectrally high-resolving analysis of the incoming speech stream is performed at the same time but in different locations, i.e. in a different mix of cell assemblies than the analysis of speaker-dependent features (such as pitch, periodicity, or other features inherent to voice quality).

Further spatially high-resolution brain imaging studies are needed to quantify as to which extent voice-selective areas in the upper bank of the STS [31] become involved when speaker categorization is accomplished. For the time being, this study increases our understanding of speech sound processing, as it replicates previous findings of an orderly mapping of phonological vowel features and as it shows that changing attentional foci affect the absolute but not the relative distribution of vowel-evoked activity within the auditory cortex.

## Methods

### Subjects

22 subjects (11 females, mean age 24.3 ± 4 years, M ± SD) participated in the procedure. All subjects were monolingual native speakers of German. Only right-handers as ascertained by the Edinburgh Handedness Questionnaire [36] were included. Subjects gave written informed consent and were paid €10 for their participation.

### Experimental design

**Figure 1:**
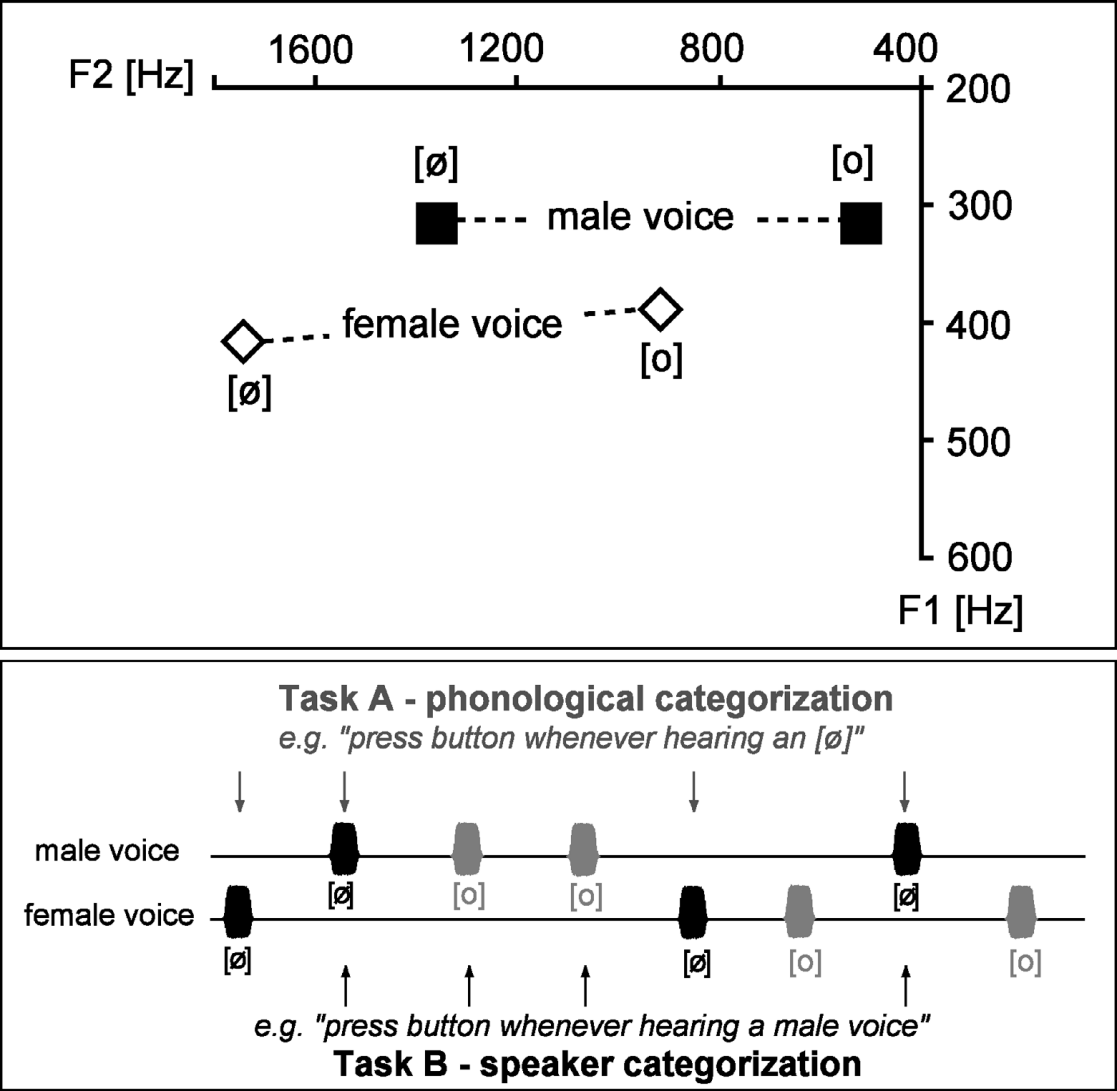
Upper panel: Illustration of the F1-F2 formant space for the vowel tokens used. Lower panel: Illustration of the stimulation paradigm and of the two tasks which all subjects performed. Attention was either focused on vowel category changes (Task A) or on changes in the voice speaking (Task B). Arrows indicate required button presses.

In an auditory target detection task, subjects listened to randomized sequences of four German natural vowel exemplars: The DORSAL rounded vowel [o] in two exemplars, in one spoken by a male voice and in the other by a female voice, and the CORONAL rounded vowel [ø], also produced by both voices (Fig. 1). 200 ms long vowels free of formant transitions were cut out of spoken words, digitized with a 10 kHz sampling rate and faded with 50 ms Gaussian on- and offset ramps. Table 1 summarizes exact pitch and formant frequencies of the four exemplars. Prior to the measurement, individual hearing thresholds were determined for both ears and all four vowel exemplars. Stimuli were presented binaurally with at least 50 dB SL (respective to the vowel exemplar which showed the weakest sensation level, if any differences between exemplars occurred) via a non-magnetic echo-free stimulus delivery system with almost linear frequency characteristic in the critical range of 200–4000 Hz.

In a test sequence, subjects repeated vowels aloud and recognized all stimuli correctly, i.e. they distinguished between both vowel categories and voices without difficulty. Binaural loudness was slightly re-adjusted where necessary to ensure perception in the head midline.

In the actual measurement, vowel exemplars were presented in two randomized sequences with equal probability and a randomized stimulus onset asynchrony of 1.6 – 2 s. All subjects performed – in a counterbalanced order – two different tasks during these two sequences: In a task A (hereafter called *phonological categorization*), subjects had to press a button with their right index finger whenever a given vowel ([o] or [ø], counterbalanced across subjects) occurred, irrespective of the speaking voice. In a task B (hereafter called *speaker categorization*), subjects had to press a button whenever a given voice (the male or the female voice, counterbalanced across subjects) uttered a vowel, irrespective of the uttered vowel category. Fig.1 (lower panel) which clarifies and visualizes the task.

That is, in the phonological categorization task, subject's attention was focused on a categorical distinction between speech sounds, [o] or [ø], which closely resembles the tasks applied in most brain imaging studies testing active speech sound processing (e.g. [14,15,37]) – a process ubiquitously taking place when decoding running speech. In contrast, the speaker categorization task was intended to shift subject's attention to more general and more basic acoustic properties of the material [31] presented to accomplish speaker distinction.

### Data reduction and statistical analyses

Data acquisition and analysis, including source modeling, closely followed the procedure described in [15]: Auditory magnetic fields were recorded using a whole head neuromagnetometer (MAGNES 2500, 4D Neuroimaging, San Diego) in a magnetically shielded room (Vaccumschmelze, Hanau, Germany). Epochs of 800 ms duration (including a 200 ms pre-trigger baseline) were recorded with a bandwidth from 0.1 to 200 Hz and a 687.17 Hz sampling rate. If the peak-to-peak amplitude exceeded 3.5 pT in one of the channels or the co-registered EOG signal was larger than 100 μV, epochs were rejected. Button-presses did not affect the auditory evoked field topography in the N100m time range.

We analyzed up to 150 artifact-free vowel responses that remained for both vowel categories [o] and [ø] after off-line noise correction, and averaged them separately for vowel category but across speaker voice. Splitting up vowel conditions into male and female speaker sub-conditions was not possible due to a resulting small number of averages. However, we also performed separate averages and analyses of male and female speaker across vowel categories. In any case, the resulting averages thus contained brain responses to two acoustically variant exemplars which makes results more comparable to our previous studies [15,17]. A 20 Hz lowpass filter (Butterworth 12 dB/oct, zero phase shift) was subsequently applied to the averages.

**Figure 2:**
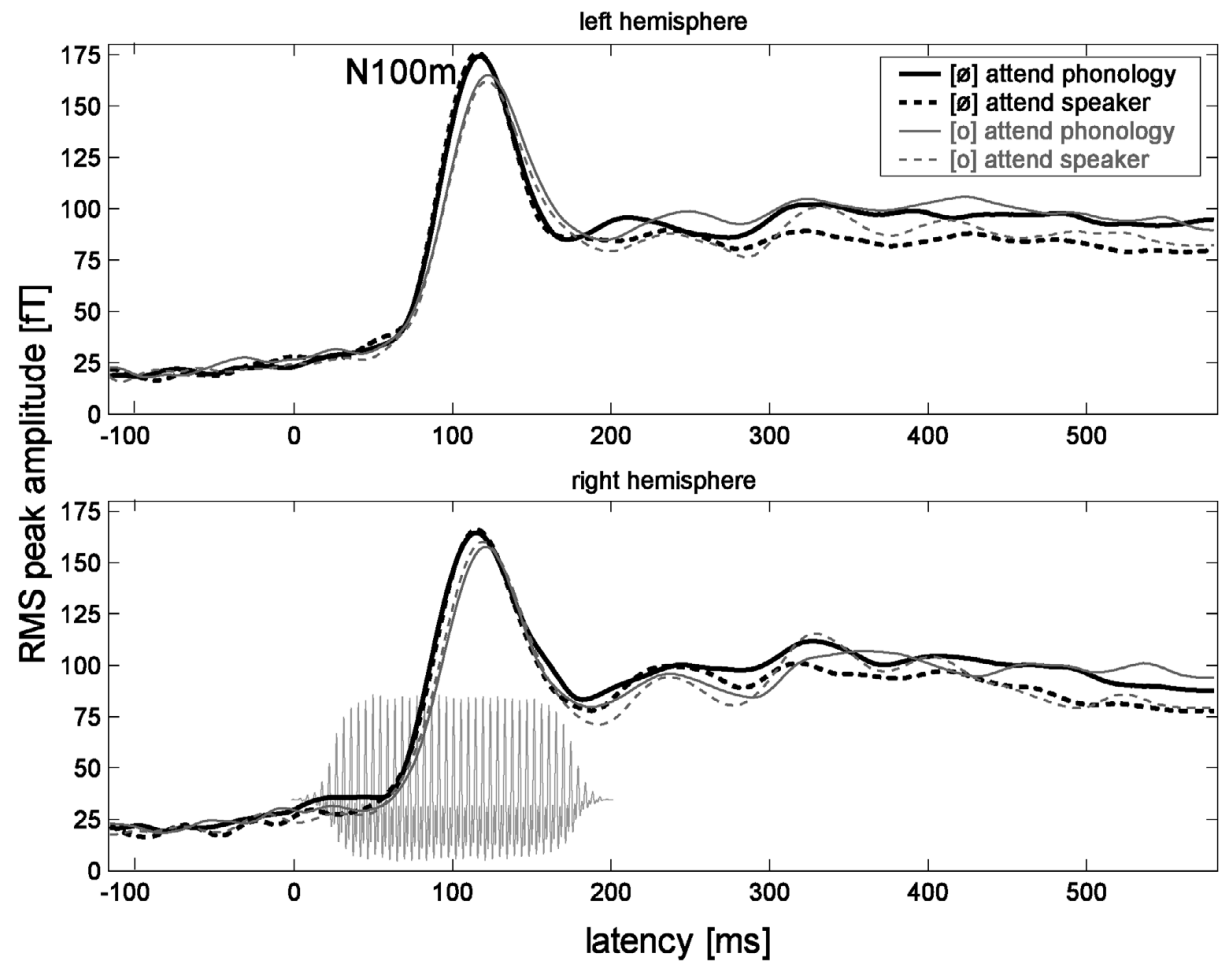
Grand average (N = 21) of root mean squared amplitudes for all conditions over time separately for left (upper panel) and right hemisphere (lower panel). N100m is clearly the most prominent waveform deflection, and the repeatedly reported N100m time lag between coronal vowel [ø] (black) and dorsal vowel [o] (gray) is also obvious.

**Figure 3:**
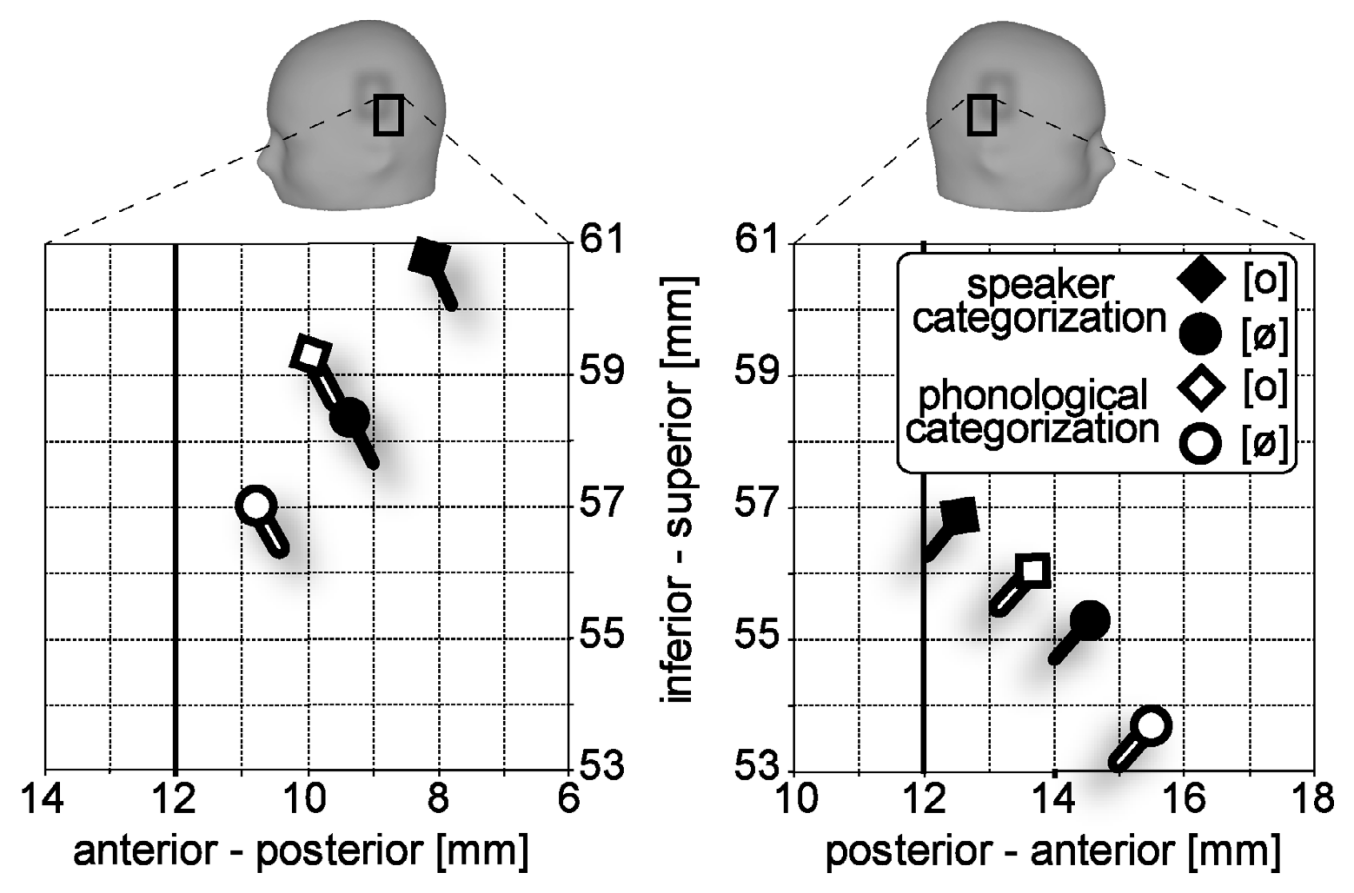
Mean two-dimensional source space locations and orientations separately for the left and the right hemisphere (posterior-anterior on abscissa, inferior-superior on ordinate) are shown. Results of the phonological categorization task are shown in open source symbols, results of the speaker categorization task in filled source symbols. Please note that in both conditions the [ø] source (circle symbols) is more inferior and anterior than the [o] source (diamond symbols).

The N100m component was defined as the prominent waveform deflection in the time range between 90 and 160 ms (Fig. 2). Isofield contour plots of the magnetic field distribution were visually inspected to ensure that N100m and not P50 m or P200 m were analyzed.

N100m peak latency was defined as the sampling point in this latency range by which the first derivative of the Root Mean Square (RMS) amplitude reached its minimum and second derivative was smaller than zero. RMS was calculated across 34 magnetometer channels selected to include the field extrema over the left and the right hemisphere, respectively.

Prior to statistical analyses, all brain response latencies were corrected for a constant sound conductance delay of 19 ms in the delivery system. Using the same sets of channels, an equivalent current dipole (ECD) in a spherical volume conductor (fitted to the shape of the regional head surface) was modeled at every sampling point separately for the left and the right hemisphere [38]. The N100m source parameters were determined as the median of 5 successive ECD solutions in the rising slope of the N100m. The resulting ECD solution represents the center of gravity for the massed and synchronized neuronal activity. To be included in this calculation, single ECD solutions had to meet the following criteria: (i) Goodness of fit greater than .90, (ii) ECD location larger than 1.5 cm in medial-lateral direction from the center of the brain and 3–8 cm in superior direction, measured from the connecting line of the pre-auricular points. Statistical analysis of dependent variables N100m peak latency, amplitude and N100m source generator strength, location and orientation focused on 2 × 2 × 2 repeated measures analysis of variance with repeated factors hemisphere (left vs. right), vowel ([o] vs. [ø]) and task (attend phonology vs. attend speaker).

As source location displacements do not appear exactly and exclusively along the Cartesian axes of the source space (cf. [21]), we additionally calculated differences in the polar angle Φ and the azimuth angle θ which here describe angular displacements in the sagittal and the axial plane, respectively.

## Authors' contributions

J.O., T.E. and C.E. conceived the experiment and drafted the manuscript. J.O. and C.E. prepared the exact experimental setup. J.O. supervised data acquisition, and performed all data and statistical analyses. All authors read and approved the final manuscript.

## Figures and Tables

**Table 1:** Formant Frequency Overview. Pitch (F_0_), formant frequencies (F_1_, F_2_, F_3_) and -distance (F_2_-F_1_) for the vowels used.

Vowel	voice	**F_0 _**(Hz)	**F_1 _**(Hz)	**F_2 _**(Hz)	**F_3 _**(Hz)	**F_2_-F_1 _**(Hz)
**[o]**	**male**	123	317	516	2601	199
**[ø]**	**male**	123	318	1357	1980	1039
**[o]**	**female**	223	390	904	2871	514
**[ø]**	**female**	223	417	1731	2627	1314
